# Reconstructing Risk Dimensions in Telemedicine: Investigating Technology Adoption and Barriers During the COVID-19 Pandemic in Taiwan

**DOI:** 10.2196/53306

**Published:** 2025-02-03

**Authors:** Tzu-Chi Wu, Chien-Ta Ho

**Affiliations:** 1 Graduate Institute of Technology Management National Chung-Hsing University Taichung Taiwan; 2 Department of Emergency Medicine Show Chwan Memorial Hospital Changhua Taiwan; 3 College of Health Sciences Central Taiwan University of Science and Technology Taichung Taiwan

**Keywords:** telemedicine, perceived risk, technology acceptance model, risk, performance risk

## Abstract

**Background:**

The COVID-19 pandemic has shifted health care toward virtual and online models, impacting both users and providers. Numerous user concerns and perceived risks related to telemedicine are continually evolving and adjusting in response to the pandemic. In many countries, there has been a substantial increase in the use of virtual health care visits, which offers a unique opportunity for researchers to explore these user concerns.

**Objective:**

This study aimed to first reconstruct the risk dimensions associated with telemedicine, then identify the risk factors affecting users’ adoption, and finally propose effective solutions to mitigate these concerns. By integrating the newly constructed perceived risk with the technology acceptance model (TAM), we scrutinized various dimensions of perceived risk and their influence on users’ perceptions of ease of use, perceived usefulness, and use intention (UI).

**Methods:**

Our target population consists of adults aged ≥18 years who have used or may use telemedicine services, recruited through an anonymous, voluntary, open, web-based survey. We collected responses and used part of them to reconstruct risk dimensions using exploratory factor analysis. Subsequently, we analyzed the intricate relationship between perceived risk, the TAM, and the acceptance of telemedicine using structural equation modeling with another part of the responses.

**Results:**

A total of 1600 valid responses were collected. Eight distinct risk dimensions were reconstructed, revealing a substantial negative impact of performance risk on UI. The psychological and social risk was the strongest barrier to the ease of using telemedicine. Time risk, provider risk, and privacy risk were not statistically significant to the TAM. The resulting model elucidates a remarkable 66% variance in UI for telemedicine services.

**Conclusions:**

This study substantially advances the field of telemedicine research by reconstructing and redefining 8 risk dimensions and confirming the statistical significance of 5 perceived risks on the adoption of telemedicine services. These insights are poised to facilitate the promotion and enhancement of telemedicine services in the health care sector.

## Introduction

### Background

During the COVID-19 crisis, telemedicine gained global prominence and became more accessible and applicable than ever before [1]. Social distancing measures necessitated the use of telemedicine as a substitute for in-person health care interactions in many countries. Health care users and providers, who had previously been reluctant to adopt telemedicine, were compelled to embrace it [2]. A substantial disruption such as a pandemic can act as a catalyst for the acceptance of telemedicine services, potentially leading to a shift in users’ perceptions and attitudes [3]. Consequently, the current expansion of telehealth has provided an opportunity to explore and reevaluate the concepts of technology acceptance and risk.

Telemedicine is an umbrella term defined as the use of technology to deliver health care and health information to individuals who are located at a distance from health care providers [4]. While the concept of telemedicine is not new, its wide-scale adoption began in the 1990s. However, telehealth is currently operating within a more information-rich environment. Due to the rapid development of telecommunication technologies and the high cost of devices, its adoption continues to face various challenges, particularly in certain regions where the full benefits of telemedicine services have not yet been realized [5,6].

According to Chen et al [7], the impact of technology on the development of telemedicine in Taiwan began with the National Information Infrastructure project, which used various network bandwidths for telemedicine starting in 1995. Before the legalization of telemedicine through an amendment to the Physician Act in 2018, Taiwan’s medical laws restricted its practice to remote areas, mountainous regions, and outlying islands [8]. Following this amendment, the Taiwan Ministry of Health and Welfare expanded the nationalized health insurance program to include coverage for telemedicine outpatient consultations across different specialties in remote areas. Despite the technological advancements, use of telemedicine remains limited in Taiwan. Reports indicate a disappointingly low rate of use, with only approximately 5.83% of individuals quarantined at home in Hsinchu City using telemedicine consultations from February to May 2020 [8]. With the onset of the COVID-19 pandemic, the government further promoted telemedicine services, resulting in a larger pool of virtual visits. This expansion provides researchers with a valuable opportunity to better understand user concerns. During the COVID-19 pandemic, telemedicine in Taiwan was primarily used for treating confirmed COVID-19 cases and chronic diseases care. Due to quarantine and social distancing requirements, patients used mobile phones, tablets, or computers to engage in telemedicine consultations with health care professionals from the comfort of their homes.

Telemedicine service is not only a technological innovation but also a sociocultural phenomenon [5]. It transforms the traditional physician-patient relationship, altering both its complexity and essence [5]. A systematic review of telemedicine emphasizes the need for more patient-oriented studies, recognizing it as a complex collaborative process [9]. In addition, Bashshur et al [5] highlight the lack of research on the social, cultural, and psychological dimensions of telemedicine.

### This Study

This study aims to approach the impact of risk from psychological and social perspectives, comprehending its influence on telemedicine, and proposing strategies for development, implementation, and acceptance [5].

Perceived risk is a substantial barrier to the adoption of telemedicine services, encompassing social and psychological aspects [10]. Bakshi and Tandon [11] introduced and validated a theory-driven framework that uncovers the facets of perceived risk as barriers to telemedicine acceptance in their study involving physicians in north India. Harst et al [12] argued that user-centered design and theory-guided approaches are crucial in telemedicine development. The impact of risk on the adoption of a technology is undeniable. However, with the increasing stream of literature, many different dimensions of risk have been proposed. Some studies adopt a limited number of risk dimensions, while others introduce risk dimensions from other fields; some risks share similar concepts or have a high degree of overlap. First, there is a shortage of effective categorization and definition among the various risks. Second, there remains a lack of research that comprehensively assesses the multiple dimensions of risk in telemedicine. Therefore, this study addresses substantial gaps in the telemedicine literature related to perceived risk facets and provides solutions. We aim to reconstruct the effective dimensions of perceived risk and explore what primarily influences users’ acceptance of telemedicine services. We seek to evaluate the integration of the technology acceptance model (TAM) into the perceived risk dimensions, providing a solid theoretical foundation for telemedicine acceptance during a crisis.

Therefore, we posed the following research questions: (1) What are the evidence-based effective risk dimensions for telemedicine services adoption? and (2) Which risks effectively influence users’ intention to use telemedicine during the COVID-19 pandemic?

To address these questions, we will conduct a comprehensive literature review to identify potential risk types in telemedicine from existing literature, providing a strong theoretical foundation. We will then empirically reconstruct these risk dimensions through exploratory factor analysis (EFA) [13]. By applying confirmatory factor analysis (CFA), we will validate and redefine the risk factors identified in the literature. Subsequently, we will integrate these redefined risk dimensions with the TAM and use CFA to explore the relationship between the 2, aiming to provide robust and credible results to strengthen the theoretical framework.

### Theoretical Background

#### TAM Overview

The TAM was designed as a model of users’ acceptance of information technologies and systems in 1986, and 3 years later, it was used to understand the attitude of computer users [14,15]. The final version of the TAM, introduced by Venkatesh and Davis [16] in 1996, eliminated the construct of attitude and found that both perceived ease of use (PEOU) and perceived usefulness (PU) positively influence use intention (UI).

The TAM model included constructs of PU, PEOU, and UI. PU was defined as the degree of users’ belief that using a particular system or service would enhance their performance [17]. PEOU means the degree of their belief that using a particular system or service would be free from effort [17]. TAM theorizes that PU and PEOU are antecedents to the adoption of technology determined by external factors [17]. In prior studies on telemedicine, PEOU positively affects PU, and both PU and PEOU positively affect UI [17].

The TAM has been used to explain various forms of computer technology and is also one of the most applied theories in the field of telemedicine [12]. Many studies considered that the TAM is an appropriate model that can be adapted to the study of technology acceptance and use of telemedicine [18,19]. One systematic review by Harst et al [12] indicates that the adaption of telemedicine service can be evaluated by using the theories of TAM and acceptance is predicted by PU, social influences, and attitude. In our study, we used the TAM to evaluate the adoption of telemedicine and hypothesized that it would be influenced by perceived risk.

#### Perceived Risk

#### Overview

The perceived risk was developed from the psychological theory proposed by Bauer [20] in 1960 to explore consumer behavior. The perceived risk is defined as a combination of uncertainty plus seriousness of the outcome involved [20]. Featherman and Pavlou [21] also adopted the same definition. Perceived risk refers to an individual’s subjective expectation of experiencing a loss in pursuit of the desired outcome [21,22]. Perceived risk consists of individuals’ subjective feelings and the self-evaluation of the size of the potential loss [23]. Thereafter, researchers increasingly applied perceived risk to consumer behavior and many dimensions of perceived risk were identified [21,24,25].

Cunningham [23] identified performance and psychosocial elements as perceived risk’s 2 major categories and typified perceived risk into 6 dimensions, such as performance, safety, financial loss, time, and social and psychological loss [23]. Then, the importance of privacy concerns has been gradually recognized and the rich literature on consumer risk perception appeared [21]. Provider risk, physical risk, and technology risk have been presented to better understand the adoption of e-services [21]. The fear that has stemmed from the COVID-19 crisis has recently caused psychological changes in terms of consumer behavior. Information on the objective risk of the pandemic is typically scarce and is featured as unpredictable and uncertain. It remains unclear how people perceive infection risks and whether these perceptions influence their decision to adopt telemedicine [26,27].

Perceived risk has been a prominent subject of discussion within the realms of telemedicine and health care in general. In Table 1, we present a comprehensive review of previous studies that have incorporated perceived risk or its related dimensions as variables in telemedicine research, using structural equation modeling (SEM) as the primary analytical approach. The literature on perceived risk suggests different types of risk, which can be flexibly applied according to the characteristics of the product or service [28]. Among these studies, only the work of Bakshi and Tandon [11] attempts to understand the barriers to telemedicine adoption by exploring different facets of perceived risk. It is worth noting that not all risk dimensions have been included in SEM models or assessed for patients in prior studies. However, a substantial number of studies investigating the relationship between telemedicine and perceived risk did not adopt SEM as their chosen research methodology.

In the subsequent sections, we will define each risk construct and provide ample justification for selecting these constructs through the existing literature, supported by theoretical backing. Furthermore, we will reconstruct the risks using EFA and obtain empirical evidence with CFA. This approach will be used to reconstruct the effective risk dimensions and validate the reliability and influence of these risk constructs on UI.

Ten dimensions of risk were selected based on our literature review. We will discuss their relationship with telemedicine in the subsequent sections.

**Table 1:** Previous literature about perceived risk and telemedicine using structural equation modeling (SEM).

Study, year	Participants	PR^a^	FNR^b^	TMR^c^	PFR^d^	PLR^e^	SCR^f^	PRR^g^	PSR^h^	TNR^i^	PVR^j^	COR^k^
Rho et al [29], 2015	Users									✓	✓	
Kondrateva et al [30], 2020	Students	✓						✓				
Kamal et al [10], 2020	Users	✓						✓				
Baudier et al [31], 2021	Users	✓										
Esmaeilzadeh and Mirzaei [32], 2021	Users					✓		✓				
Kaphzan et al [33], 2022	Psychiatrists	✓										
Rad et al [34], 2022	Providers										✓	
Bakshi and Tandon [11], 2022	Physicians		✓	✓			✓	✓		✓		
Xiao et al [35], 2023	Users		✓	✓				✓				
Liu et al [36], 2024	Users	✓										
Bahari et al [37], 2024	Users			✓		✓		✓				
Singh et al [38], 2024	Older users		✓			✓				✓		

^a^PR: perceived risk.

^b^FNR: financial risk.

^c^TMR: time risk.

^d^PFR: performance risk.

^e^PLR: psychological risk.

^f^SCR: social risk.

^g^PRR: privacy risk.

^h^PSR: physical risk.

^i^TNR: technology risk.

^j^PVR: provider risk.

^k^COR: COVID-19 infection risk.

#### Financial Risk

The financial risk refers to potential monetary loss owing to transaction errors or subsequent maintenance costs of the productor service [11]. Financial risk in telemedicine pertains to insurance, billing, payment system, and equipment costs. Historically, insurance coverage and reimbursement for telemedicine services has been a major barrier to adoption [39]. Before the COVID-19 pandemic, most commercial health insurance plans restricted the types of services covered, and many excluded telehealth services. Users were worried that insurance payment regulations did not allow the coverage of telemedicine, which would cause extra loss of money [39]. Second, the lack of awareness about appropriate service charges and the way of payment is another cause of risk. Because of payment restrictions and complex and diversified billing before the pandemic, users’ fear about the perceived risk of monetary loss was caused by transaction errors, though some countries had legalized payment parity for telehealth and more user-friendly payment systems have been developed as of now. Third, the purchasing and maintenance cost of the equipment for telemedicine and as well as the internet is relatively high, particularly in developing countries [39]. Topacan et al [40] opined that users and designers of health services should be concerned about the cost of telemedicine services, which should be even less than the cost of visiting a physician [40]. These factors lead to the following concerns: (1) using telemedicine (video consultations) might involve additional costs (financial risk 1); (2) uncertainty about the fees for telemedicine worries me (financial risk 2); (3) complex online payment systems during telemedicine could lead to financial losses (financial risk 3); and (4) uncertainty regarding insurance coverage during telemedicine might result in financial setbacks (financial risk 4).

#### Performance Risk

A performance risk is the potential that a product or service will not deliver as much value and desired benefits as required [25]. Inconsistent service quality and failure to meet expectations contribute to perceived risk and consumer dissatisfaction [41]. Generally, the content of the service should be related to the physicians’ professionalism and users’ requirements. Deviations from one’s expectations of care may result in reduced satisfaction [42]. Topacan et al [40] stated that users attach importance to obtaining accurate, relevant, and quality information in a minimum response time. A cross-sectional survey of 699 German mayors identified misdiagnosis and differences in disease applications as the primary perceived risks of telemedicine [43,44]. Users are concerned that not only would the same patient-physician connection not be established but they would not receive the level of complex care they may require because diseases could not be diagnosed through telemedicine services compared with face-to-face consultations [45,46]. These reviews and observations lead to the following survey questions: (1) telemedicine services might not be able to address my concerns (performance risk 1); (2) telemedicine might not meet my expected standards (performance risk 2); and (3) the effectiveness of telemedicine might not compare to in-person consultations (performance risk 3).

#### Technology Risk

Technology risk can be defined as a technological failure that disrupts business and increases the users’ perceived uncertainty about telemedicine technology. Bakshi and Tandon [11] revealed that technology risk had a negative effect on a group of physicians’ intention to adopt telemedicine. Consumers are reported to have the same concern. The most frequently cited barriers among patients on dialysis who use home telehealth services are malfunctioning equipment or trouble with internet connections [47]. In addition, a cross-sectional survey in Australia revealed that factors including ineffective communication, the lack of scope for physical examination, and difficulty obtaining prescriptions or examination results were reasons for perceived risk. Technological limitation is the reason why user’s telehealth experiences were poorer than traditional face-to-face consultation experiences [46]. Hence, the survey includes the following questions: (1) I worry that the equipment for telemedicine might not function properly (technology risk 1); (2) I am concerned that physicians might not gather my information thoroughly during telemedicine consultations (technology risk 2); (3) I fear the absence of physical examinations (such as touch, percussion, or auscultation) during telemedicine services (technology risk 3); (4) I am worried about the effectiveness of communication with the physician during telemedicine consultations (technology risk 4); and (5) I am concerned about difficulties in obtaining medications or learning about test results through telemedicine services (technology risk 5).

#### Psychological Risk

Perceived psychological risk refers to users’ perception of any possible psychological frustration or anxiety resulting from the use of telemedicine services, which is relatively complicated and with which users are unfamiliar compared to long-term face-to-face consultation [48]. Thus, users may operate the service system unsuccessfully, which can cause anxiety and psychological pressure, called technology anxiety. The term is derived from studies on computer anxiety in which users hold anxious and negative attitudes toward computers [49]. Previous studies among physicians in Ethiopia have shown that computer systems can bring about anxiety; users tend to have an emotional response and become anxious when they are requested to use the service [50]. The following questions are based on these observations: (1) using telemedicine services might make me anxious (perceived psychological risk 1); (2) using telemedicine services might make me feel uncomfortable (perceived psychological risk 2); (3) using telemedicine services might create stress for me, such as worrying about technical errors (perceived psychological risk 3); and (4) using telemedicine services might negatively impact my self-image and self-esteem (perceived psychological risk 4).

#### Social Risk

Featherman and Pavlou [21] defined social risk as the “potential loss of status in user’s social group as a result of adopting the service, looking foolish, or untrendy” [21]. Social risk and influence may come from subjective norms, social factors, and specific interpersonal agreements [40]. People are influenced by others’ opinions and behaviors. Individuals have favorable or unfavorable perceptions of telemedicine that in turn affects their perspectives; alternatively, not adopting telemedicine may also have positive or negative connotations [51]. Harst et al [12] demonstrated that not only the target users but the user’s social environment should be considered when planning telemedicine service. This is because of the importance of users’ social environment and because individuals’ social group can support their use of telemedicine. One worldwide study showed that peer experience is one of the major factors behind the overall adoption of telemedicine, as it seems more attractive to them after seeing their family and friends successfully using it. The following questions are based on these observations: (1) using telemedicine service might lower my self-esteem (social risk 1); and (2) using telemedicine service might lead my family and friends to have negative opinions about me (social risk 2).

#### Physical Risk

Physical risk means the risk of potential threat to users’ safety, physical health, or well-being. Physical risk in telemedicine services is related to the safety and health of individuals within high-risk environments [25,52]. During the COVID-19 pandemic, telemedicine could help mildly ill patients to receive the medical care they need at home while minimizing their exposure to the coronavirus [53]. Therefore, the survey contains the following questions: (1) using telemedicine service might pose risks to my physical health (physical risk 1); (2) using telemedicine service might harm my well-being (physical risk 2); and (3) using telemedicine service might lead to illness or infectious diseases, such as pneumonia (physical risk 3).

#### Privacy Risk

Privacy risk refers to the possibility of the loss of private data owing to fraud or hacking, which compromises the information security of online users [51]. Privacy concerns are a major barrier to online technology adoption [54]. Issues in telemedicine included substantial concerns about privacy and information security [55]. The users’ critical concern about privacy included the maximum protection of personal electronic records and clinical data. Transmission and storage of sensitive information must be transparent [56]. This guarantee must be provided by those who supply the device; they must ensure that the devices are safe and noninvasive so that the users do not feel they were spied on [55]. Studies in the United States suggest that people tend to trust hospitals, which substantially moderate the negative privacy effects [54]. Therefore, the survey contains the following questions: (1) I feel that my privacy might not be secure when using telemedicine service (privacy risk 1); (2) I worry that if health care facilities do not have proper storage and backup, my personal data might be lost during the use of telemedicine service (privacy risk 2); (3) I am concerned that my personal information or medical condition might be leaked during telemedicine service (privacy risk 3); and (4) I fear that my personal data might be stolen or misused during telemedicine service (privacy risk 4).

#### Provider Risk

Provider risk refers to the degree to which users perceive uncertainty about telemedicine providers, including companies, hospitals, organizations, primary care providers, and physicians [29]. A nationwide survey in the United States emphasizes the importance of providers, stating that respondents become less willing to use telemedicine as they become further detached from their own providers [57]. On the basis of another nationwide survey involving 4345 adults in the United States, most participants expressed hesitation when it came to using telemedicine for consultations with health care providers not affiliated with their current organization. Only a mere 19% indicated a willingness to use it for a provider from an entirely different organization [58]. Therefore, the survey contains the following questions: (1) I worry that the providers of telemedicine service might not be knowledgeable (provider risk 1); (2) I am concerned whether professional medical personnel provide this telemedicine service (provider risk 2); and (3) I fear that the providers (physicians or hospitals) might not have enough capability during telemedicine service (provider risk 3).

#### Time Risk

Time risk is defined as the loss of time and inconvenience caused by delays in finding appropriate services and in learning to use particular technology [11,21]. Most users do not prefer using medical services that demand more time [40]. During the process, consumers may waste time learning how to use telemedicine services and are forced to switch to face-to-face consultation if it does not meet their expectations. Moreover, in developing economies, inadequate provision of information and communication technology infrastructure and poor internet connectivity are still formidable barriers leading to the wastage of time [57]. In contrast, a worldwide study showed that one of the major factors behind the overall adoption of telemedicine is that it saves time, as telemedicine can reduce physical travel and queue times at the hospital [59]. In addition, in some countries, telemedicine visits usually take less time than in-person visits, which are substantially associated with higher levels of satisfaction [60]. So, the survey contains the following questions: (1) accepting telemedicine service might require additional time (time risk 1); (2) slow internet speeds could potentially waste my time (time risk 2); and (3) If telemedicine service do not meet expectations, it might lead to even more time wastage (time risk 3).

#### Perceived COVID-19 Infection Risk

There are 2 dimensions in perceived COVID-19 infection risk: the cognitive and affective dimension and the dimension related to fear and general concerns. The cognitive or affective dimension is the qualitative dimension in the perception of COVID-19, such as its severity, controllability, and personal impact. However, the dimension related to general concerns focuses on personal feelings of fear and insecurity. Perceived risk related to COVID-19 does not come under the perceived risk of telemedicine; however, a crisis situation such as the COVID-19 pandemic influences the perceptions of telemedicine [59]. Following these theories, we can expect that people with higher risk perceptions are more likely to adopt telemedicine services. Therefore, the survey includes the following questions: (1) I feel my health is at risk during the COVID-19 pandemic (perceived COVID-19 infection risk 1); (2) I believe it is challenging to control the COVID-19 outbreak (perceived COVID-19 infection risk 2); (3) I worry about severe harm to my body if I contract the virus (perceived COVID-19 infection risk 3); (4) I think the current COVID-19 situation is more serious than before (perceived COVID-19 infection risk 4); and (5) I fear I might get infected by the coronavirus (perceived COVID-19 infection risk 5).

## Methods

### Instrument Development

The target population in our study were users and potential users of telemedicine services in Taiwan. The questionnaire for the survey was adopted from previous telemedicine studies or studies on fields related to health care. Both constructs suggested by the TAM model and perceived risk dimensions were included in the questionnaire [21]. To improve its quality and clarity, the questionnaire’s preliminary version was tested to a pilot group of 60 citizens through the web-based survey and 3 individuals holding master’s degree suggested modifications in the wording of items for conciseness. Finally, some questions were deleted owing to their low α reliability. The measurement scales were assessed on a 5-point Likert scale—from 1 for “strongly disagree” to 5 for “strongly agree.” Then, it was determined whether the value of Cronbach α in all constructs was >0.65, which meant that the internal consistency and reliability of summated rating scales were acceptable [61].

### Ethical Considerations

We conducted a noninterventional questionnaire-based study, using social science methods to gain a deeper understanding of participants’ perceptions. According to the Taiawn Ministry of Health and Welfare's Human Research Act, Article 5, research conducted in public settings involving nonspecific individuals, which is noninterventional, anonymous, and where the collected information cannot be used to identify specific individuals, is exempt from review [62]. At the initial stage of questionnaire distribution, participants were informed of the study’s purpose and made aware that their participation was voluntary and anonymous, with no personal or private data being collected. All procedures were conducted in accordance with the ethical guidelines of the Declaration of Helsinki.

### Data Collection

The study uses SurveyCake, a widely used and professional questionnaire distribution platform in Taiwan. Our target population includes individuals who have previously used telemedicine services or those who may potentially use them in the future. Recruitment was restricted to adults aged ≥18 years, as they possess medical decision-making autonomy. We primarily used public social media platforms, such as Facebook (Meta Platforms, Inc), to reach a broad audience. To ensure the representativeness of our sample, we extended our outreach to relevant online communities across various geographical regions, aiming to achieve balance in terms of age and urban-rural distribution. In addition, for populations less familiar with the internet, we used local community networks to raise awareness about recruitment. By engaging participants through a diverse range of online and offline platforms, we aimed to ensure that the sample accurately reflected Taiwan’s current population, particularly in terms of sex, age, education level, and regional distribution.

### Data Management

After the questionnaire collection is complete, we conducted preliminary processing of the results, including the deletion of incomplete responses. In addition, some questionnaires deemed to have low reliability were removed, including those with reverse order questions and extreme outlier values (such as very short response times and selecting the same option for all questions). Next, the collected questionnaires were randomly divided into 2 groups: group A and group B, using simple random sampling in SPSS (version 20; IBM Corp). Subsequently, normality tests were conducted to determine whether the datasets approximated a normal distribution by testing the normality of the data using the Kolmogorov-Smirnov residual method with the appropriate criteria.

The exported data were managed by SPSS and the relationship between variables was evaluated with SPSS Amos (version 26). SEM is the preferred technique among many standard methods in software and enables the evaluation of complex, multiple latent constructs and relationships [63]. Moreover, because the study analyzes the cause and different facets of perceived risk and its effect on technology acceptance, SEM has been preferred over regression. SEM comprises 2 parts, EFA and CFA. The questionnaires from group A, used for EFA, were used to ensure that the variables adequately represented the domains of the factors and to exclude variables from unrelated domains, thereby reconstructing several evidence-based risk dimensions. Meanwhile, the questionnaires from group B were used for CFA to validate the hypothesized model and its paths [64].

### EFA, Construct Extraction, and Definition

First, the questionnaires from group A were used to justify the application of EFA by accessing the Bartlett test of sphericity with Kaiser-Meyer-Olkin, which was conducted with Varimax rotation and the results of EFA [65,66]. Next, we redesigned the facets of perceived risk based on eigenvalues exceeding 1.000, as determined by CFA. The EFA aims to extract domains with eigenvalues greater than 1.000, and to name the newly generated dimensions based on a combination of data analysis results (such as factor loadings) and theoretical background. In this step, we reconstructed latent variables of perceived risk, and informed the hypothesis according to the abovementioned literature review.

### Bias, Reliability, and Validity

To address common method bias (CMB), we conducted a Harman 1-factor test, which involved performing an EFA on all study variables. The results indicated that the first factor accounted for >50% of the variance among the variables [67]. To mitigate concerns of multicollinearity, we used the variance inflation factor to evaluate the potential bias generated by the measurement design and determine the degree of multicollinearity among the multiple regression variables, with a suggested threshold of <10 [68].

To assess the validity of the model, 2 types of validities were used: convergent and discriminant validities. The convergent validity reflects the extent to which 2 measures capture a common construct [69]. Convergent validity is estimated by using the factor loading of all indicators and the average variance extracted (AVE). If the value of the factor loading of all items is >0.50, it is considered substantial. In addition, if the AVE is >0.5 and composite reliability is not <0.7, it indicates adequate convergence [70]. Moreover, the discriminant validity reflects the degree to which items are discriminated against amid constructs or individual conceptions are assessed for the measurement model. Two criteria are used for the estimation of discriminant validity. The first one is that the AVE itself is greater than correlations in a given construct’s column or row [71]. The second one is that the square root of AVE of the corresponding construct exceeds any correlation with another construct, which means the discriminant validity is considered to be established [72].

### Model Fit

To ensure the fit between the data and the measurement model or to assess the goodness of fit, 4 different measures were used, including chi-square—the ratio of the chi-square statistic to the respective *df* (*χ*^2^/*df*), the goodness of fit index (GFI), root mean square error of approximation, and the comparative fit index. The chi-square is an absolute fit index, with a low chi-square value relative to the df indicating a better model fit. However, the chi-square fit statistic is affected by large samples and a ratio is <5 for the *χ*^2^/*df* is preferred for acceptable model fit evaluation [73-75].

### Hypothesis Testing

After the measurement model was confirmed, we used the structural model to explain the relationship among the variables and discover the connection between constructs followed by hypothesis testing. Then, bootstrapping resampling technique was used to examine model paths in the structural model—the *P* values <.05 were considered statistically significant [76].

## Results

### Overview

The web-based survey of our study was open to the public in Taiwan from June 13 to 30, 2022. A total of 1638 responses to the questionnaire were received, of which 38 were deleted owing to incompletion or low reliability caused by short use of answer time. Therefore, 1600 valid responses were collected for the analysis with a valid response rate of 98.2%. Using simple random sampling, all the questionnaires were divided into 2 groups: group A (600 questionnaires, 37.5%) and group B (1000 questionnaires, 62.5%). Table 2 reports the characteristics of all respondents in more detail, including reasonable representations of sex, age, and educational qualifications. Normality was assessed using the 1-sample Kolmogorov-Smirnov test, with significance values for group A and group B recorded as 0.200 and 0.550, respectively. Because both values exceed 0.05, we conclude that the data are normally distributed.

**Table 2:** Sample demographic of the respondents.

Characteristics		Group A (n=600)	Group B (n=1000)	Total (N=1600)
**Sex, n (%)**				
	Male	301 (50.1)	474 (47.4)	775 (48.4)
	Female	299 (49.9)	526 (52.6)	825 (51.6)
**Age (y), n (%)**				
	18-29	213 (35.5)	318 (31.8)	531 (33.2)
	30-39	116 (19.3)	169 (16.9)	285 (17.8)
	40-49	98 (16.4)	214 (21.4)	312 (19.5)
	>50	173 (28.8)	299 (29.9)	472 (29.5)
**Education level, n (%)**				
	High school	71 (11.8)	129 (12.9)	200 (12.5)
	Bachelor	357 (59.5)	607 (60.7)	964 (60.3)
	Master	155 (25.8)	241 (24.1)	396 (24.7)
	Doctoral	17 (2.8)	23 (2.3)	40 (2.5)
**Experience of telemedicine, n (%)**				
	None	510 (85)	839 (83.9)	1349 (84.3)
	1-3 times	85 (14.1)	147 (14.7)	232 (14.5)
	>3 times	5 (0.9)	14 (1.4)	19 (1.2)
**History of chronic disease, n (%)**				
	None	477 (79.5)	801 (80.1)	1278 (79.8)
	Yes	123 (20.5)	199 (19.9)	322 (20.2)

Thus, group A were used to justify the application of EFA by accessing the Bartlett test of sphericity with Kaiser-Meyer-Olkin value was 0.925 and the Bartlett test of sphericity was significant (*P*<.001) [65,66]. The test was conducted with Varimax rotation and the results of EFA indicated that 8 different factors, with eigenvalues being superior to 1.000, accounted for 69.9% of the cumulative variance. Technology risk 1, technology risk 2, technology risk 4, and perceived COVID-19 infection risk 3 were deleted owing to lower factor loading, wrong concept of contrast, or the crossing of 2 different contrasts during the EFA process.

**Figure 1:**
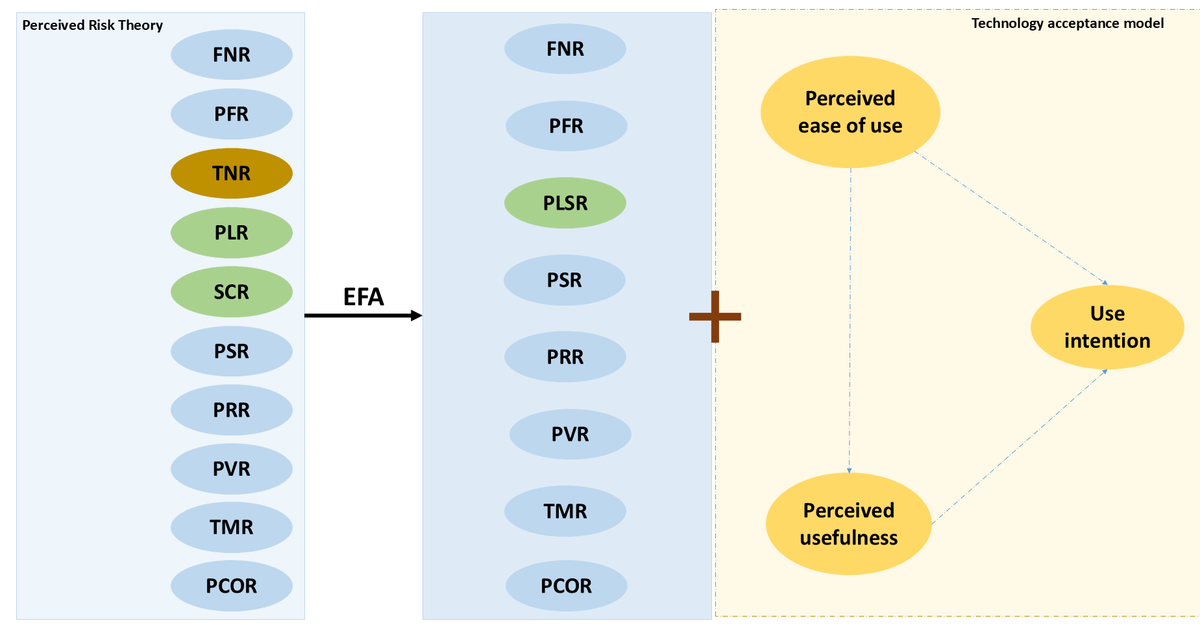
Eight risk dimensions reconstructed through exploratory factor analysis (EFA). FNR: financial risk; PCOR: perceived COVID-19 infection risk; PFR: performance risk; PLR: psychological risk; PLSR: psychological and social risks; PSR: physical risk; PRR: privacy risk; PVR: provider risk; SCR: social risk; TMR: time risk; TNR: technology risk.

According to the results of EFA, 2 risk facets, namely “technology risk” and “social risk,” were removed from the analysis. In addition, the facet originally labeled as “perceived psychological risk” was renamed “psychological and social risks” because the 2 items related to social risk were classified under the same category as perceived psychological risk by the EFA. As a result, a total of 8 facets related to perceived risk were identified (Figure 1) and details including factor loading are presented in Table 3. To sum up, 8 risk dimensions were reconstructed, including financial risk, performance risk, psychological and social risk, physical risk, privacy risk, provider risk, time risk, and perceived COVID-19 infection risk.

**Table 3:** Varimax with Kaiser normalization.

Factor and item		Factor loading of components							
		1	2	3	4	5	6	7	8
**FNR^a^**									
	FNR1	0.702	—^b^	—	—	—	—	—	—
	FNR2	0.737	—	—	—	—	—	—	—
	FNR3	0.704	—	—	—	—	—	—	—
	FNR4	0.649	—	—	—	—	—	—	—
**PFR^c^**									
	PFR1	—	0.828	—	—	—	—	—	—
	PFR2	—	0.822	—	—	—	—	—	—
	PFR3	—	0.767	—	—	—	—	—	—
	TNR3	—	0.610	—	—	—	—	—	—
**PLSR^d^**									
	PLR1	—	—	0.741	—	—	—	—	—
	PLR2	—	—	0.823	—	—	—	—	—
	PLR3	—	—	0.689	—	—	—	—	—
	PLR4	—	—	0.676	—	—	—	—	—
	SCR1	—	—	0.611	—	—	—	—	—
	SCR2	—	—	0.596	—	—	—	—	—
**PSR^e^**									
	PSR1	—	—	—	0.800	—	—	—	—
	PSR2	—	—	—	0.823	—	—	—	—
	PSR3	—	—	—	0.714	—	—	—	—
**PRR^f^**									
	PRR1	—	—	—	—	0.660	—	—	—
	PRR2	—	—	—	—	0.775	—	—	—
	PRR3	—	—	—	—	0.865	—	—	—
	PRR4	—	—	—	—	0.833	—	—	—
**PVR^g^**									
	PVR1	—	—	—	—	—	0.639	—	—
	PVR2	—	—	—	—	—	0.691	—	—
	PVR3	—	—	—	—	—	0.583	—	—
	TNR5	—	—	—	—	—	0.589	—	—
**TMR^h^**									
	TMR1	—	—	—	—	—	—	0.743	—
	TMR2	—	—	—	—	—	—	0.777	—
	TMR3	—	—	—	—	—	—	0.587	—
**PCOR^i^**									
	PCOR1	—	—	—	—	—	—	—	0.721
	PCOR2	—	—	—	—	—	—	—	0.813
	PCOR4	—	—	—	—	—	—	—	0.756
	PCOR5	—	—	—	—	—	—	—	0.714

^a^FNR: financial risk.

^b^Not applicable.

^c^PFR: performance risk.

^d^PLSR: psychological and social risks.

^e^PSR: physical risk.

^f^PRR: privacy risk.

^g^PVR: provider risk.

^h^TMR: time risk.

^i^PCOR: perceived COVID-19 infection risk.

### Definition of Factors of Risk

The definitions of the 8 facets of perceived risk and constructs of TAM are provided in Table 4. The 8 risk dimensions included financial risk, performance risk, psychological and social risk, physical risk, privacy risk, provider risk, time risk, and perceived COVID-19 infection risk.

**Table 4:** The definition of the constructs.

Constructs	Operational definition	Reference
Financial risk	Potential for financial loss due to online payments, limited insurance coverage, and maintenance costs for products like mobile phones and the internet.	[39,77]
Performance risk	The result does not match the desired benefits.	[21,48,78]
Psychological and social risks	The risk that lowers the user’s self-image, leads to a loss of self-esteem and embarrassment, and generates pressure and anxiety resulting from the use of a service.	[79]
Physical risk	The risk to the user’s safety and physical health in using telemedicine.	[78]
Privacy risk	Potential loss of control over personal health information without permission.	[77,80]
Provider risk	The risk of the user’s perceived uncertainty about the telemedicine provider.	[29,81]
Time risk	The risk of the user’s perception of wastage of time in learning how to use the service.	[21,77]
Perceived COVID-19 infection risk	The fear and general concerns about people’s perception of COVID-19 infection.	[82]
Perceived ease of use	The degree of users’ belief that using telemedicine service would be free from effort.	[83]
Perceived usefulness	The degree of users’ belief that using telemedicine service would enhance their performance.	[83]
Use intention	Users’ desire to use telemedicine services in the future.	[10,77]

### Hypothesis Formation

According to the aforementioned literature review, 18 hypotheses were derived (Textbox 1).

**Derived hypotheses.**

- Hypothesis 1a: financial risk negatively influences the perceived ease of use (PEOU) of telemedicine services.
- Hypothesis 1b: financial risk negatively influences the usage intention (UI) in telemedicine services.
- Hypothesis 2a: performance risk negatively influences the PEOU in telemedicine services.
- Hypothesis 2b: performance risk negatively influences the perceived usefulness (PU) of telemedicine services.
- Hypothesis 2c: performance risk negatively influences the UI in telemedicine services.
- Hypothesis 3a: psychological and social risks negatively influences the PEOU of telemedicine services.
- Hypothesis 3b: psychological and social risk negatively influences PU in telemedicine services.
- Hypothesis 3c: psychological and social risk negatively influences UI in telemedicine services.
- Hypothesis 4a: physical risk negatively influences the PU of telemedicine services.
- Hypothesis 4b: physical risk negatively influences the UI in telemedicine services.
- Hypothesis 5: privacy risk negatively influences the UI in telemedicine services.
- Hypothesis 6: provider risk negatively influences the UI in telemedicine services.
- Hypothesis 7: time risk negatively influences the UI in telemedicine services.
- Hypothesis 8a: perceived COVID-19 infection risk positively influences the PU of telemedicine services.
- Hypothesis 8b: perceived COVID-19 infection risk positively influences the UI in telemedicine services.
- Hypothesis 9: PEOU positively influences the PU in telemedicine services.
- Hypothesis 10: PEOU positively influences the UI in telemedicine services.
- Hypothesis 11: PU positively influences the UI in telemedicine services.

### Research Model

**Figure 2:**
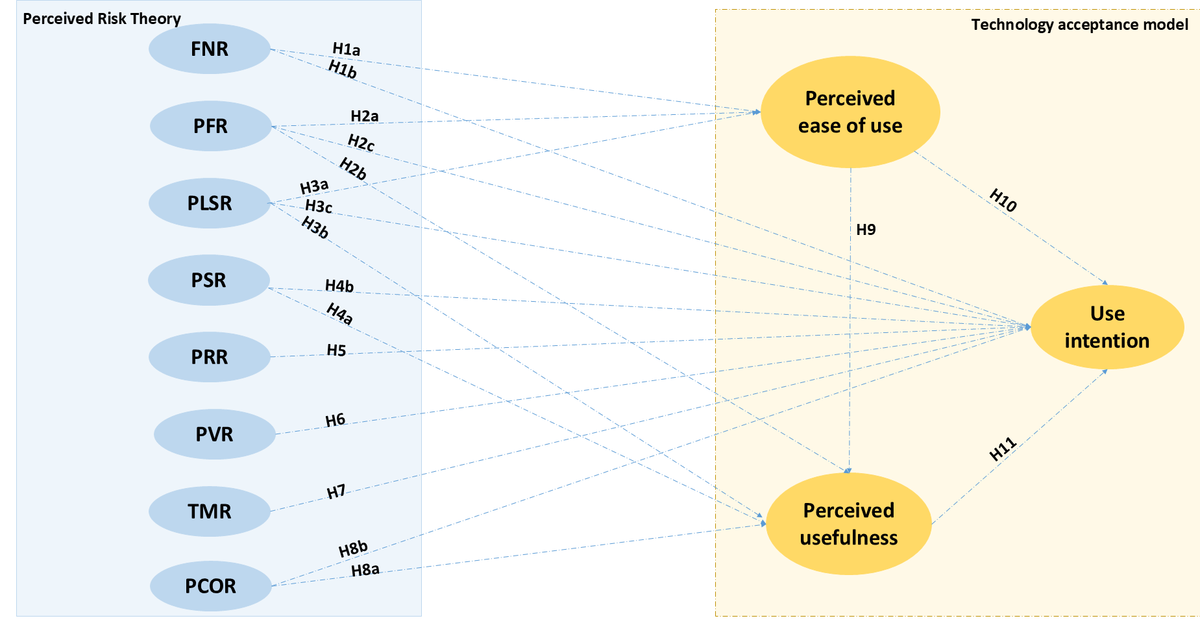
Research model and hypothesis. FNR: financial risk; PFR: performance risk; PLSR: psychological and social risks; PSR: physical risk; PRR: privacy risk; TMR: time risk.

The research model (Figure 2) consists of 8 redefined dimensions of risk combined with the TAM, resulting in a total of 18 hypotheses.

### Reliability, Validity, and Bias

In CFA for the reliability and validity test (Table 5), financial risk 1 and perceived COVID-19 infection risk 5 were deleted owing to lower standard loading. PU1 was deleted from the subsequent analysis according to the result of modification indices because of a higher correlation with UI and failure of the test of discriminant validity (the square root of the AVE for PU 0.715 is less than PU of the correlations with UI 0.766). After that, the tests indicate adequate discriminant validity. Two criteria regarding discriminant validity showed that not only all AVEs were greater than the correlations in that construct’s rows and columns but also the square root of AVE (diagonal) was greater than off-diagonal elements in rows and columns. The two tests mentioned earlier support discriminant validity (Table 6).

In addition, the result of the evaluation showed that all Cronbach α values were >0.7 and the internal consistency of items was considered. Moreover, all item loads on their own factors were >0.5 and the internal consistency reliability for each construct was >0.6. Most constructs’ AVE values met the minimum requirement because the constructs exceeded the criterion of 0.50, though the construct—“perceived COVID-19 infection risk”—was 0.394 and “financial risk” was 0.483. However, we could accept AVE<0.5 if the composite reliability was >0.6 because the convergent validity of the construct was still adequate. Therefore, these tests indicate adequate convergent validity [72].

Financial risk, performance risk, psychological and social risk, physical risk, privacy risk, provider risk, time risk, and perceived COVID-19 infection risk had mean scores of 3.33 (SD 0.854), 3.58 (SD 0.783), 2.43 (SD 0.769), 2.49 (SD 0.897), 3.39 (SD 1.00), 3.32 (SD 0.878), 3.37 (SD 0.831), and 3.81 (SD 0.751), respectively. The mean scores of predictors of TAM such as PU, PEOU, and UI, were 4.00 (0.761), 3.81 (SD 0.804), and 3.87 (SD 0.768), respectively.

The values of variance inflation factor for all the latent variables in the model were between 1.107 and 2.241, all of which were lower than the suggested threshold, indicating low concerns about multicollinearity. For addressing CMB, the Harman 1-factor test during the EFA on all study variables showed the first factor accounted for 31% of the variance among the variables, which was lower than the suggested threshold, indicating no CMB issues.

**Table 5:** Reliability, validity test, and means among latent constructs.

Items		Standard loading	Values, mean (SD)		AVE^a^	CR^b^	Cronbach α
**FNR^c^**			3.33 (0.854)		0.483	0.780	0.807
	FNR2	0.727					
	FNR3	0.776					
	FNR4	0.791					
**PFR^d^**			3.58 (0.783)		0.599	0.853	0.845
	PFR1	0.842					
	PFR2	0.880					
	PFR3	0.765					
	TNR3	0.571					
**PLSR^e^**			2.43 (0.769)		0.613	0.905	0.902
	PLR1	0.781					
	PLR2	0.825					
	PLR3	0.733					
	PLR4	0.807					
	SCR1	0.799					
	SCR2	0.749					
**PSR^f^**			2.49 (0.897)		0.632	0.835	0.819
	PSR1	0.827					
	PSR2	0.906					
	PSR3	0.626					
**PRR^g^**			3.39 (1.00)		0.702	0.903	0.897
	PRR1	0.685					
	PRR2	0.811					
	PRR3	0.930					
	PRR4	0.903					
**PVR^h^**			3.32 (0.878)		0.585	0.847	0.840
	PVR1	0.755					
	PVR2	0.823					
	PVR3	0.849					
	TNR5	0.609					
**TMR^i^**			3.37 (0.831)		0.505	0.749	0.745
	TMR1	0.543					
	TMR2	0.745					
	TMR3	0.816					
**PCOR^j^**			3.81 (0.804)		0.394	0.716	0.705
	PCOV1	0.569					
	PCOV2	0.764					
	PCOV4	0.673					
**PU^k^**			4.00 (0.761)		0.655	0.792	0.791
	PU2	0.799					
	PU3	0.821					
**PEOU^l^**			3.81 (0.751)		0.703	0.904	0.904
	PEOU1	0.841					
	PEOU2	0.812					
	PEOU3	0.843					
	PEOU4	0.857					
**UI^m^**			3.87 (0.768)		0.656	0.884	0.882
	UI1	0.849					
	UI2	0.811					
	UI3	0.747					
	UI4	0.828					

^a^AVE: average variance extracted.

^b^CR: critical ratio.

^c^FNR: financial risk.

^d^PFR: performance risk.

^e^PLSR: psychological and social risks.

^f^PSR: physical risk.

^g^PRR: privacy risk.

^h^PVR: provider risk.

^i^TMR: time risk.

^j^PCOR: perceived COVID-19 infection risk.

^k^PU: perceived usefulness.

^l^PEOU: perceived ease of use.

^m^UI: use intention.

**Table 6:** Correlation matrix of variables.

	1	2	3	4	5	6	7	8	9	10	11
FNR^a^	0.695	—^b^	—	—	—	—	—	—	—	—	—
PFR^c^	0.473	0.774	—	—	—	—	—	—	—	—	—
PLSR^d^	0.468	0.443	0.783	—	—	—	—	—	—	—	—
PSR^e^	0.388	0.387	0.658	0.795	—	—	—	—	—	—	—
PRR^f^	0.571	0.548	0.471	0.433	0.838	—	—	—	—	—	—
PVR^g^	0.672	0.584	0.520	0.450	0.773	0.775	—	—	—	—	—
TMR^h^	0.643	0.687	0.350	0.294	0.531	0.579	0.711	—	—	—	—
PCOR^i^	0.213	0.185	0.108	0.093	0.214	0.204	0.235	0.627	—	—	—
PU^j^	–0.184	–0.342	–0.365	–0.294	–0.220	–0.234	–0.260	0.086	0.810	—	—
PEOU^k^	–0.335	–0.306	–0.483	–0.323	–0.279	–0.325	–0.249	0.029	0.607	0.838	—
UI^l^	–0.205	–0.460	–0.356	–0.341	–0.256	–0.280	–0.311	0.046	0.775	0.625	0.810

^a^FNR: financial risk.

^b^Not applicable.

^c^PFR: performance risk.

^d^PLSR: psychological and social risks.

^e^PSR: physical risk.

^f^PRR: privacy risk.

^g^PVR: provider risk.

^h^TMR: time risk.

^i^PCOR: perceived COVID-19 infection risk.

^j^PU: perceived usefulness.

^k^PEOU: perceived ease of use.

^l^UI: use intention.

### Model Fit

**Figure 3:**
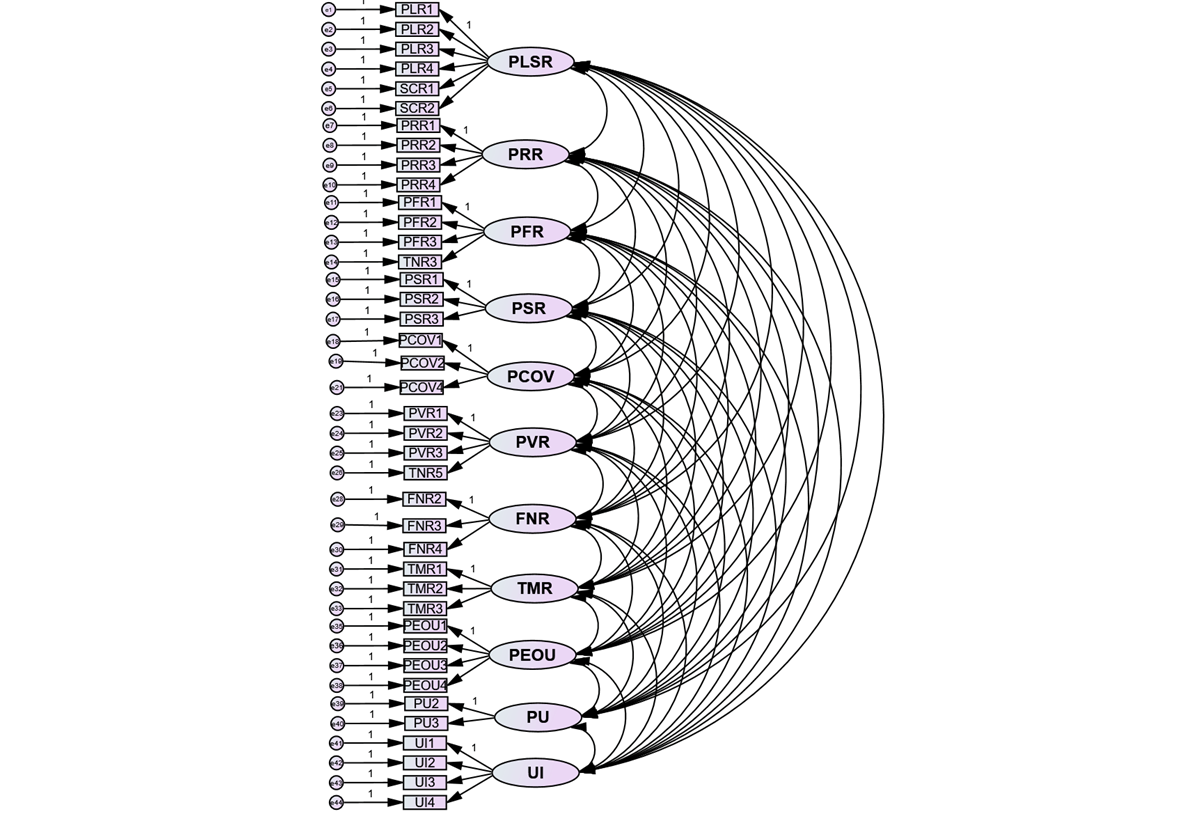
Confirmatory factor analysis (CFA) for the measurement model. FNR: financial risk; PCOR: perceived COVID-19 infection risk; PEOU: perceived ease of use; PFR: performance risk; PLSR: psychological and social risks; PRR: privacy risk; PU: perceived usefulness; PVR: provider risk; TMR: time risk; UI: use intention.

In the following procedure, the measurement model (Figure 3) assessment was done, and a summary of the model fit measurements is presented in Table 7, in which most results surpass the acceptable levels but the value of GFI is 0.840 which is below 0.9. As Doll et al [84] suggest, GFI in the 0.8-0.9 range presents a reasonable fit, particularly for more variance [84]. Overall, the measurement model indicates a satisfactory fit.

**Figure 4:**
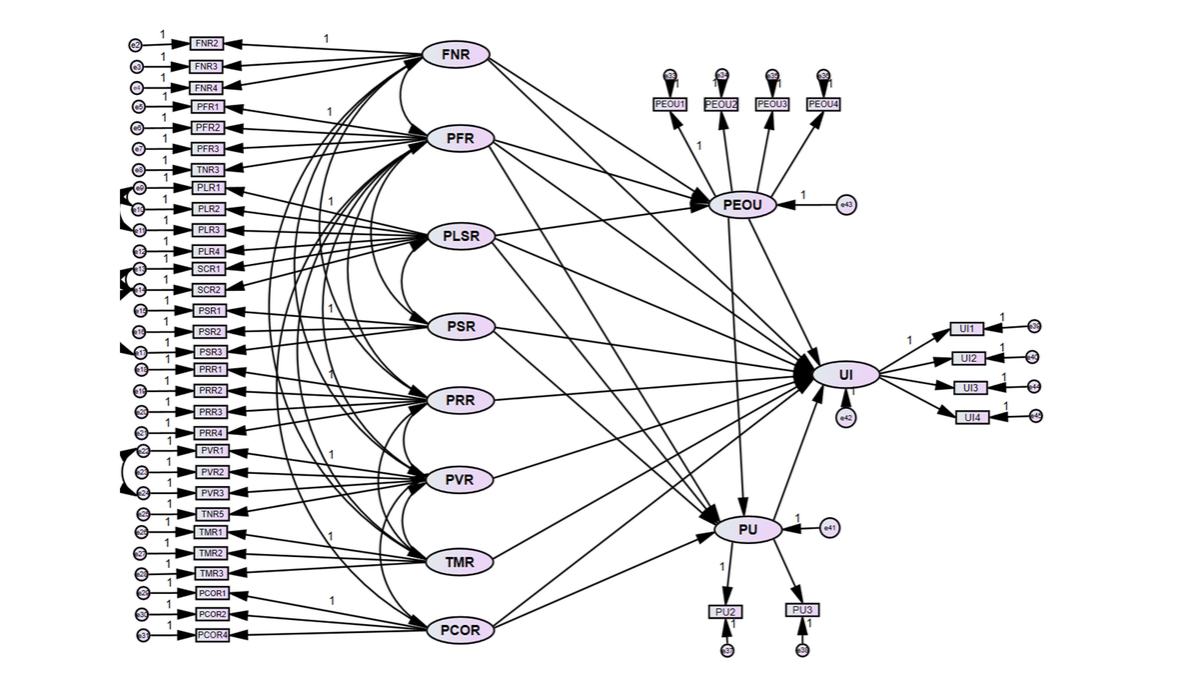
The structural model. FNR: financial risk; PCOR: perceived COVID-19 infection risk; PEOU: perceived ease of use; PFR: performance risk; PLSR: psychological and social risks; PRR: privacy risk; PU: perceived usefulness; PVR: provider risk; TMR: time risk; UI: use intention.

After the measurement model was confirmed, we used the structural model (Figure 4) to discover how closely the theory is supported by empirical data and whether the theory is empirically confirmed [85]. The result of the structural model and the summary of the model fit measurement are given in Table 8. The structural model indicates a satisfactory fit. We also tested for second-order factors in multifaceted risk perception. However, the model fit indices were inferior to those of the first-order model. Therefore, we decided to retain the original structural model.

**Table 7:** Fit measures in the structural model.

Fit measure	Measurement model	Recommended values
*χ*^2^ (*df*)	3116 (685)	—^a^
*χ*^2^/*df*	4.55	<5
Goodness of fit index	0.840	>0.9
Comparative fit index	0.902	>0.9
Root mean square error of approximation	0.060	<0.08

^a^Not available.

**Table 8:** Results of hypotheses testing.

Fit measure	Structural model	Recommended values
*χ* ^2^ *(df)*	2908 (703)	—^a^
*χ* ^*2*^ */df*	4.136	<5
Goodness of fit index	0.869	>0.9
Comparative fit index	0.911	>0.9
Root mean square error of approximation	0.056	<0.08

^a^Not available.

### Result of Hypotheses

**Figure 5:**
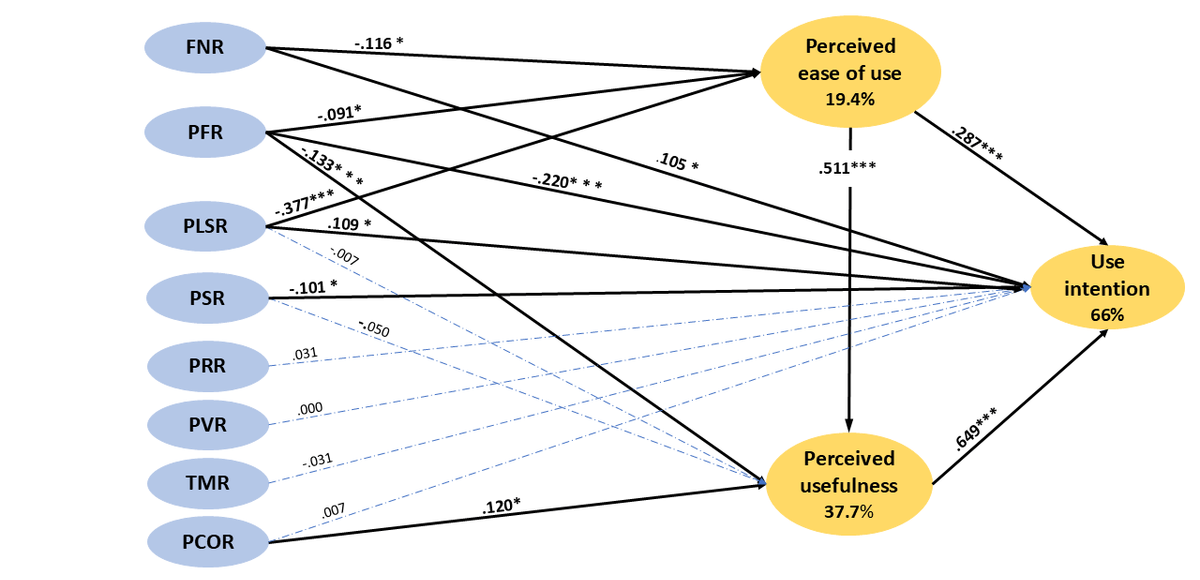
Results of hypotheses testing. FNR: financial risk; PFR: performance risk; PLSR: psychological and social risks; PSR: physical risk; PRR: privacy risk; PVR: provider risk. *P<.05, ***P<.001.

The hypothesis testing showed that 5 out of 8 redefined risks significantly affect the TAM. The result of the hypotheses testing is shown in Table 9. Three kinds of perceived risk—financial risk, performance risk and psychological and social risk—had significant negative effects on PEOU. The psychological and social risk showed a stronger effect (β=−0.377; *P*<.001) than the financial risk (β=−0.116; *P*=.003) and performance risk (β=−0.091; *P*=.006). PU was affected by 2 types of perceived risk. The performance risk (β=−0.133; *P*<.001) had a significant negative effect, but the perceived COVID-19 infection risk (β=0.120; *P*=.003) had a significant positive effect on PU. Furthermore, performance risk was the only kind of risk that had a significant direct negative impact on all 3 variables: PEOU, PU, and UI. However, time risk, provider risk, and privacy risk did not significantly impact the 3 contrasts of TAM. PU was significantly influenced by PEOU (β=0.511; *P*<.001). Moreover, both PU and PEOU were found to significantly affect UI (*P*<.001). In terms of goodness of fit indicators, the research model explained 66% of the variance in UI of telemedicine service as shown in Figure 5. To further assess the significance of the direct and indirect effects of predictor variables on the UI of telemedicine service, a decomposition of the effects analysis was conducted in Table 10. Overall, among the 8 types of risks, performance risk had the most significant total effect on UI (β=−0.362).

**Table 9:** Results of hypotheses testing.

Hypothesis	Path coefficient (SE)	CR^a^	*P* value	Result
Hypothesis 1a: PEOU^b^<-FNR^c^	−0.116 (0.039)	−2.996	.003	Accepted
Hypothesis 1b: UI^d^<-FNR	0.105 (0.048)	2.183	.03	Accepted
Hypothesis 2a: PEOU<-PFR^e^	−0.091 (0.033)	−2.754	.006	Accepted
Hypothesis 2b: PU^f^<-PFR	−0.133 (0.029)	−4.542	<.001	Accepted
Hypothesis 2c: UI<-PFR	−0.220 (0.041)	−5.419	<.001	Accepted
Hypothesis 3a: PEOU<-PLSR^g^	−0.377 (0.038)	−9.799	<.001	Accepted
Hypothesis 3b: PU<-PLSR	−0.007 (0.048)	−0.140	.89	Rejected
Hypothesis 3c: UI<-PLSR	0.109 (0.044)	2.465	.01	Accepted
Hypothesis 4a: PU<-PSR^h^	−0.050 (0.037)	−1.359	.17	Rejected
Hypothesis 4b: UI <-PSR	−0.101 (0.034)	−2.979	.003	Accepted
Hypothesis 5: UI<-PRR^i^	0.031 (0.043)	0.717	.47	Rejected
Hypothesis 6: UI <- PVR^j^	0.000 (0.040)	0.001	<.99	Rejected
Hypothesis 7: UI <- TMR^k^	−0.031 (0.067)	−0.467	.64	Rejected
Hypothesis 8a: PU <-PCOR^l^	0.120 (0.040)	2.992	.003	Accepted
Hypothesis 8b: UI <-PCOR	0.007 (0.037)	0.190	.85	Rejected
Hypothesis 9: PU <-PEOU	0.511 (0.038)	13.319	<.001	Accepted
Hypothesis 10: UI <-PEOU	0.287 (0.042)	6.896	<.001	Accepted
Hypothesis 11: UI <-PU	0.649 (0.049)	13.281	<.001	Accepted

^a^CR: critical ratio.

^b^PEOU: perceived ease of use.

^c^FNR: financial risk.

^d^UI: use intention.

^e^PFR: performance risk.

^f^PU: perceived usefulness.

^g^PLSR: psychological and social risks.

^h^PSR: physical risk.

^i^PRR: privacy risk.

^j^PVR: provider risk.

^k^TMR: time risk.

^l^PCOR: perceived COVID-19 infection risk.

**Table 10:** Direct effects, indirect effects, and total effects.

		PFR^a^	PLSR^b^	PSR^c^	PCOR^d^	FNR^e^	PRR^f^	PVR^g^	TMR^h^	PEOU^i^	PU^j^
**PEOU**											
	Direct effect	−0.091	−0.377	—^k^	—	−0.116	—	—	—	—	—
	Indirect effect	—	—	—	—	—	—	—	—	—	—
	Total effect	−0.091	−0.377	—	—	−0.116	—	—	—	—	—
**PU**											
	Direct effect	−0.133	−0.007	−0.050	0.120	—	—	—	—	0.511	—
	Indirect effect	−0.046	−0.192	−0.048	—	−0.059	—	—	—	—	—
	Total effect	−0.179	−0.199	−0.098	0.120	−0.059	—	—	—	0.511	—
**UI**											
	Direct effect	−0.220	0.109	−0.101	0.007	0.105	0.031	—	−0.031	0.287	0.649
	Indirect effect	−0.142	−0.237	−0.033	0.078	−0.072	—	—	—	0.331	—
	Total effect	−0.362	−0.128	−0.134	0.085	0.033	0.031	—	−0.031	0.618	0.649

^a^PFR: performance risk.

^b^PLSR: psychological and social risks.

^c^PSR: physical risk.

^d^PCOR: perceived COVID-19 infection risk.

^e^FNR: financial risk.

^f^PRR: privacy risk.

^g^PVR: provider risk.

^h^TMR: time risk.

^i^PEOU: perceived ease of use.

^j^PU: perceived usefulness.

^k^Not available.

## Discussion

### Principal Findings

The study developed a theoretical model based on TAM to determine the barriers to UI in telemedicine services. By reconstructing the risk dimensions, 8 facets of perceived risk were extracted through EFA, and 5 facets of perceived risk were considered to substantially affect the TAM of telemedicine services during the COVID-19 pandemic in Taiwan. Three risk dimensions have been adjusted according to the results of EFA. Perceived psychological risk and social risk have been categorized into psychological and social risk, mainly due to their high degree of overlap. Social risk emphasizes the potential for image damage due to lack of recognition by others, while perceived psychological risk highlights the potential for inconsistency with self-identity. Social perceptions may also influence one’s self-image, as well as attitudes toward and anxiety about telemedicine. Technology risk, which is not traditionally categorized under risk perception, was included in previous studies primarily from a technological perspective to assess the impact of risks. This inclusion leads to some overlap with the original risk dimensions. For instance, survey questions asked users whether they were concerned about the poor performance of telemedicine due to various technological limitations (such as network, equipment, and physical examination). Although these are technological limitations, they fundamentally address concerns about performance. This was the purpose of EFA—to reconstruct 8 effective types of risk.

The psychological and social risk seems to be the strongest barrier to perceived risk affecting the ease of using telemedicine. Our study supports the effect of technology anxiety and pressure, which have been shown to affect users’ resistance to using new technology [10]. Technology anxiety and uncertainty can lead to resistance to change owing to unexpected errors in technology [49]. Therefore, when users are faced with a new telemedicine service, they may be reluctant to switch from a face-to-face service to an alternative one. In addition, our results agree with the past finding that psychological and social risk prevents not only providers but also users from adopting technology [11]. Users care about social pressure from their friends, family members, and work groups with regard to telemedicine service.

According to the results of the total effect, performance risk is the most substantial risk factor affecting UI when Taiwanese individuals consider the adoption of telemedicine. In addition, performance risk was the only risk factor that had a negative effect both on UI directly and on PEOU and PU at the same time. This indicates that PEOU and PU are the mediators that partially explain the association between performance risk and UI. Concerns about the performance, technology, and quality of service have been expressed by stakeholders. For both health care providers and users, performance always plays a vital role. A large number of medical studies on the effectiveness of telemedicine have compared it with traditional in-person visits with regard to different diseases, such as anxiety disorders [2] and chronic kidney diseases [86]. Variations of quality and effectiveness in telemedicine and in-person care may lead to performance risk. Another factor leading to performance risk is distrust and concern regarding the technology. As shown in the previous research, the result provides more empirically grounded evidence for the importance of technology concerns perhaps caused by inadequate communication effects between physicians and patients and by disruptions in communication owing to an unstable broadband connection, which may alter the accuracy of instructed physical examination results and even cause medical error. Therefore, the “digital divide” means that telemedicine service cannot completely substitute for face-to-face visits. Indeed, a substantial concern shared by both physicians and users is the inability to conduct an in-person physical examination [87]. In the study, the item technology risk 3, “I fear the absence of physical examinations during telemedicine services,” was classified as a factor related to performance risk during the EFA. This indicates that individuals not only have concerns about the limitations of the technology itself but also about the impact it may have and the resulting performance outcomes. As Nguyen et al [42] mentioned in the review, promoting realistic expectations before the telemedicine visit takes place is essential to effectively improve patient satisfaction [42]. Providers should try to increase technology acceptance and a sense of trust by assuring the users of various technological solutions to reduce the risk or by showing evidence for the similarity between technological use and in-person examination components through virtual assessments [88].

We found that the perceived COVID-19 infection risk has a positive effect on PU with *P* value of <.001. This is because telemedicine was globally used by force of circumstance and because of social distancing as a substitute for face-to-face interactions in enhancing clinical care, health promotion, and disease prevention. The substantial negative effect of financial risk on the PEOU of telemedicine is notable. In this context, financial risk refers to payment models, personal and governance insurance coverage, and online payment systems. One possible reason for the impact of financial risk could be the general public’s understanding of Taiwan’s nationalized health insurance program, which may not be fully aware of the extent and scope of insurance coverage for telemedicine services. In addition, users may have concerns about online transactions due to their perception of financial loss risks. Studies on online payment highlight the importance of website security signs and quality information, as these factors can influence customers’ perception of financial risk [89]. These results may underscore the importance of trust in online trading platforms and payment systems.

Three facets of perceived risk—time risk, provider risk, and privacy risk—do not appear to be statistically significant to TAM in telemedicine service. In the past, internet privacy concerns were one major barrier to the adoption of digital technologies or online services [90]. However, the absence of a substantial effect of privacy risk on UI in telemedicine in our hypotheses agrees with the findings of the study by Kamal et al [10], in which perceived privacy risk was found to be an insignificant determinant of UI to use telemedicine services. Moreover, as shown by studies among 1059 US residents, just like environmental risks similar to the COVID-19 pandemic, privacy concerns do not negatively impact UI. Another cross-sectional study in Germany also revealed that data privacy and security concerns were seen as less important in case of an emergency condition [44]. One possible explanation by Kato-Lin and Thelen [54] is that people may be involved in a different decision process when they are facing immediate and severe risks. Another explanation may be the result of a tradeoff during the crisis between the privacy concern and the perceived risk of COVID-19 infection.

In general, telemedicine services were expected to reduce the need for transportation, save travel time, and provide cost-saving for the public [87,91]. However, it is worth noting that the health care system in Taiwan is characterized by good accessibility, comprehensive population coverage, and short waiting times [92]. Compared to the study by Weibel et al found that patients using telemedicine saved an average of 438 driving miles per visit, the study also highlighted the potential risks related to time and distance [42]. In Taiwan, seeking medical services is extremely convenient, with shorter time and distance required for medical visits. These advantages make it less likely for the public to experience time risk.

Regarding provider risk, previous studies found that most patients prefer to use telemedicine with their physician with whom they have an established relationship [58]. One possible explanation for the insignificant hypothesis is that most users in Taiwan primarily used telemedicine services for COVID-19 infection diagnosis and treatment during that period. This is different from the context of general chronic disease management, where the importance of the service provider is comparatively reduced. Therefore, it is the result of tradeoff among provider risk, government policy, and perceived of COVID-19 infection. Consistent with previous research, our study found that PEOU positively influences PU, and both PU and PEOU positively impact UI. However, PU exerts a stronger influence on UI [17].

As >80% of respondents had no prior telemedicine experience, the findings are particularly relevant to nonadopters and may encourage them to consider using telemedicine. Risk perception is a dynamic and continually adjusting process. Nonadopters often perceive increased uncertainty and risks due to their lack of experience with technology, driven by unfamiliarity, fear of the unknown, and privacy concerns [93]. Therefore, these perceived risks have a greater potential for mitigation through awareness campaigns and other adjustments.

### Implications and Limitations

In the promotion of telemedicine, addressing psychological and social risk is crucial in influencing user behavior. To alleviate technology anxiety and discomfort, health care providers should prioritize the establishment of emotional bonds and genuine care, particularly when catering to older adult users engaging in mobile health services [94]. Demonstrating empathy and inclusiveness toward users, particularly those experiencing anxiety and discomfort, is essential. Swiftly resolving issues and offering efficient technical support, particularly for new services, can effectively reduce user concerns and mitigate risks associated with social origins. Encouraging users to share positive experiences contributes to diminishing social risk and generating excitement among potential users.

Regarding the main barrier to adoption, performance risk, which may stem from the public’s unfamiliarity and lack of knowledge about telemedicine, as well as the unknowns and misunderstandings surrounding its performance. According to consistent evidence from systematic reviews, the quality of care in telemedicine is comparable to, or even superior to, face-to-face consultations [2]. Governments and health care providers play a critical role in informing individuals about this evidence to alleviate concerns of potential users. Close monitoring of telemedicine service quality and providing additional education tailored to underserved users can substantially enhance adoption rates.

There are some limitations to this study. First, the web-based survey using the platform SurveyCake has a methodological limitation. The sample may produce a bias toward individuals more likely to be on the web and have internet access. Second, we focused on users in Taiwan、 and our result for perceived risk may not be representative of other countries. Furthermore, at the time of the study, approximately 80% of the population in Taiwan still did not have experience with telemedicine services, which may limit the applicability of the study’s findings. Third, this survey would benefit from longitudinal data, which represents only a single point in time and does not assess trends or tradeoffs in patients’ perceived risk over time. In future studies, an examination of user’s perceived risk at other time points will help understand the change of tradeoffs among the risks. Second, to understand the effect of experience or age difference on users’ perceived risk, future studies can recruit subgroups and compare the findings with those of our study.

### Conclusions

This study aimed to enhance previous research efforts by reconstructing the facets of perceived risk at a more granular level and further refining the nomological fit of the TAM with the perceived risk dimensions. Eight effective risk dimensions were reconstructed based on a solid review of the background literature through empirical techniques. Upon analyzing the relationships between perceived risk and the TAM, we determined that performance risk was the most substantial risk factor and substantially influenced telemedicine adoption both directly and indirectly. In addition, performance risk, financial risk, and psychological and social risk substantially impacted PEOU. These findings deepen our understanding of user perceived risk and its impact on telemedicine adoption, highlighting the need for strategies to address users’ uncertainties and concerns.

On the basis of the identified factors, including performance risk and psychological and social risk, telemedicine service providers, planners, and policy makers can design better strategies for the successful implementation and adoption of the services in countries that have not yet benefited from telemedicine. To decrease public anxiety and pressure, public awareness about the adoption process should be increased, and the willingness to embrace telemedicine should be instilled in the public as a convenient way to obtain high-quality health care services. Health care providers and governments must also improve the quality of technology and widely disseminate evidence related to the effectiveness and safety of telemedicine, which will decrease performance risk and fundamentally promote the public’s intention to use telemedicine services.

## Abbreviations

AVE: average variance extracted
CFA: confirmatory factor analysis
CMB: common method bias
EFA: exploratory factor analysis
GFI: goodness of fit index
PEOU: perceived ease of use
PU: perceived usefulness
SEM: structural equation modeling
TAM: technology acceptance model
UI: use intention
